# Long-term effects of low calcium dialysates on the serum calcium levels during maintenance hemodialysis treatments: A systematic review and meta-analysis

**DOI:** 10.1038/s41598-018-23658-y

**Published:** 2018-03-28

**Authors:** Masahiro Yoshikawa, Osamu Takase, Taro Tsujimura, Etsuko Sano, Matsuhiko Hayashi, Tsuyoshi Takato, Keiichi Hishikawa

**Affiliations:** 1 Department of iPS Cell Research & Epigenetic Medicine, Keio University School of Medicine, Tokyo, Japan; 2 Department of Physiology, Keio University School of Medicine, Tokyo, Japan; 3 Apheresis and Dialysis Center, School of Medicine, Keio University School of Medicine, Tokyo, Japan; 4 Division of Tissue Engineering, University of Tokyo Hospital, Tokyo, Japan

## Abstract

Hypercalcemia and hyperparathyroidism in patients receiving maintenance hemodialysis (MHD) can cause the progression of cardiovascular diseases (CVD) and mineral bone disorders (MBD). The KDIGO recommends the dialysates with a calcium (Ca) concentration of 1.25–1.5 mmol/L for MHD treatments, but the optimal concentration remains controversial. Here, we conducted a systematic review and a meta-analysis of seven randomized controlled trials examining a total of 622 patients to investigate the optimal concentration for MHD for 6 months or longer. The dialysates with a low Ca concentration (1.125 or 1.25 mmol/L) significantly lowered the serum Ca and raised the intact parathyroid hormone levels by 0.52 mg/dL (95% confidence interval, 0.20–0.85) and 39.59 pg/mL (14.80–64.38), respectively, compared with a high Ca concentration (1.50 or 1.75 mmol/L). Three studies showed that a low concentration was preferred for lowering arterial calcifications or atherosclerosis in different arteries, but one study showed that coronary arterial calcifications increased with a low concentration. Two studies showed contradictory outcomes in terms of MBD. Our meta-analysis showed that a dialysate with a low Ca concentration lowered the serum Ca levels in patients receiving long-term MHD, but further studies are needed to determine the optimal Ca concentration in terms of CVD and MBD.

## Introduction

It is quite important to control the levels of serum calcium (Ca), phosphate (P) and parathyroid hormone (PTH) properly in patients who undergo maintenance hemodialysis (HD) because of stage 5 chronic kidney disease (CKD). Hypercalcemia, hyperphosphatemia and hyperparathyroidism (HPT) can cause the progression of cardiovascular diseases (CVD) and CKD-mineral bone disorders (CKD-MBD)^1,2^, the former of which is the leading cause of death in patients receiving maintenance hemodialysis (HD)^3^. The serum Ca, P and PTH levels were previously controlled mainly by the administration of Ca carbonate^4^ and vitamin D, such as calcitriols^5^, but both of these medications often caused hypercalcemia followed by CVD and CKD-MBD. Now, other medications, such as non-Ca-based P-binders^6^, are available that do not raise the serum Ca level, and cinacalcet^7^ even lowers the serum Ca level, making the levels of serum Ca, P and PTH easier to control.

The levels of serum Ca and PTH are also influenced by the Ca concentrations of dialysates (dCa). The Kidney Disease: Improving Global Outcomes (KDIGO) guideline^8,9^ recommends the use of a dCa of 1.25–1.5 mmol/L for maintenance HD treatments, but the optimal concentration for patients receiving long-term treatment remains controversial. Recently, several randomized clinical studies have addressed this issue, but a consensus remains to be achieved. Here, we conducted a systematic review and a meta-analysis of randomized controlled trials (RCTs) to investigate the optimal dCa to prevent the progression of CVD and CKD-MBD during long-term conventional maintenance HD.

## Results

### Database search and profiles of studies

**Figure 1:**
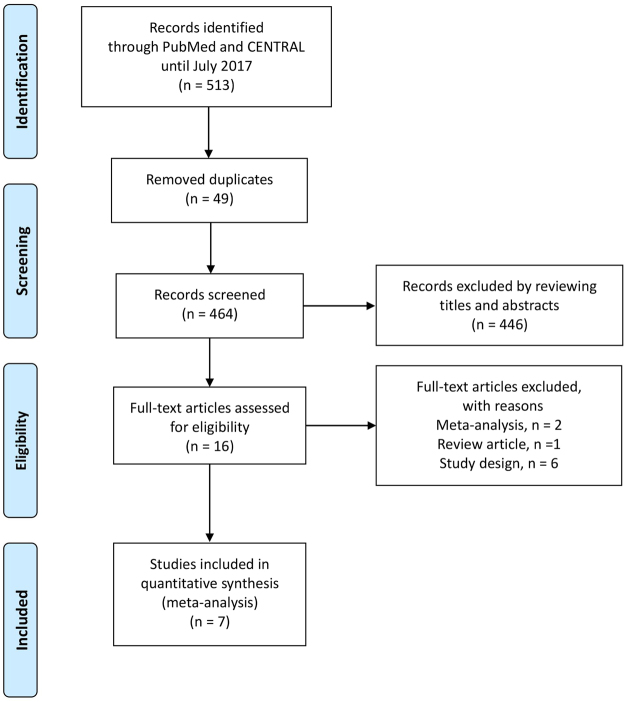
Flow diagram^17^ for our search strategy and process.

**Figure 2:**
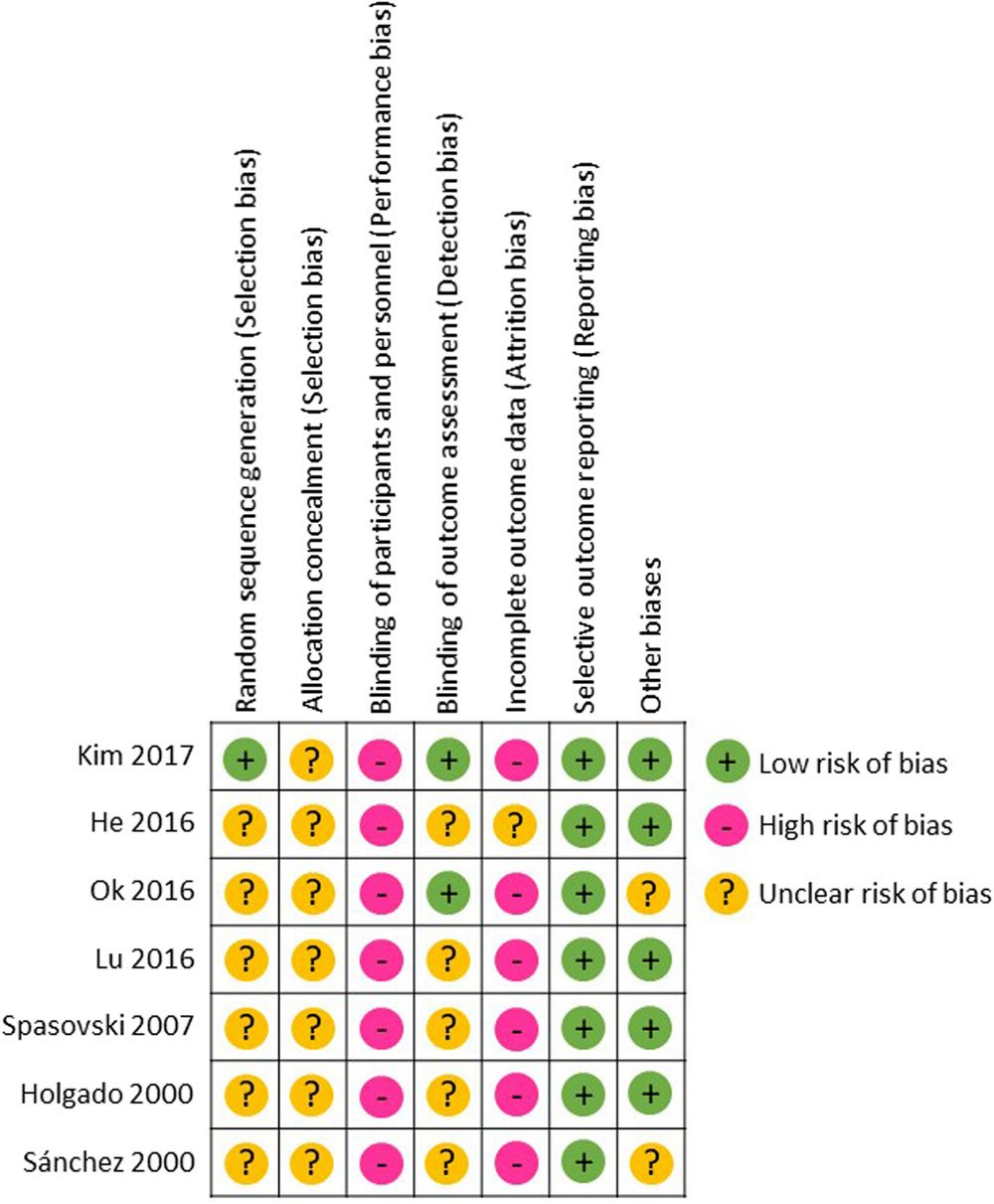
Summary of risk of bias in each included study using the Cochrane Collaboration’s tool.

We searched for studies using the PubMed and CENTRAL databases and identified a total of 513 articles. After removing duplicates, we reviewed the titles and/or abstracts and excluded 446 articles. We then assessed the full texts of the remaining 16 articles and excluded 9 articles. Finally, we adopted seven RCTs^10–16^ in which researchers compared the effects of a low Ca dialysate (LCD) of 1.25 or 1.125 mmol/L with those of a high Ca dialysate (HCD) of 1.5 or 1.75 mmol/L when used for 6 months or longer in patients undergoing conventional maintenance HD. A flow diagram^17^ showing our search strategy and process is presented in Fig. 1. The profiles of the seven included studies are shown in Table 1. A summary of the risk of bias for each of the included studies was determined using the Cochrane Collaboration’s tool^18^, as shown in Fig. 2.

**Table 1:** Characteristics and Ca, P, iPTH outcomes of included RCTs.

Author, year	Dialysate Ca (mmol/L)	Number of patients	Baseline characteristics of patients	Period of study	Medications used during study	Serum Ca (mean ± SD)	Serum P (mean ± SD)	Serum iPTH (pg/mL) (mean ± SD)
Kim, 2017	LCD = 1.25 HCD = 1.50	LCD = 30 HCD = 34	age > 20 years, Ca ≥ 8.0 mg/dL, iPTH ≤ 300 pg/mL	12 months	Ca carbonate, sevelamer, hydrochloride calcitriol	LCD = 8.4 ± 0.7 mg/dL, HCD = 8.6 ± 0.6 mg/dL, corrected Ca, p = 0.3812	LCD = 5.4 ± 1.8 mg/dL, HCD = 4.4 ± 1.6 mg/dL, p = 0.411	LCD = 151.0 (114.6 to 198.9), HCD = 71.6 (52.2 to 98.1), mean (95% CI), p = 0.0007
He, 2016	LCD = 1.25 HCD = 1.50	LCD = 59 HCD = 51	age 18 to 70 years	24 months	Ca salts, active vitamin D3	LCD = 9.07 ± 0.85 mg/dL, HCD = 9.58 ± 0.90 mg/dL, not corrected Ca, p < 0.05	LCD = 5.92 ± 1.73 mg/dL, HCD = 6.40 ± 1.67 mg/dL, n.s.	LCD = 250 (182–402), HCD = 192 (110–319), median (IR), n.s.
Ok, 2016	LCD = 1.25 HCD = 1.75	LCD = 150 HCD = 132	age 18 to 80 years, Ca ≤ 10.2 mg/dL, iPTH ≤ 300 pg/mL	24 months	Ca–based P-binders, calcitriol or alfacalcidol	values not available, shown as time-averaged data during 24 months	values not available, shown as time-averaged data during 24 months	values not available, shown as time-averaged data during 24 months
Lu, 2016	LCD = 1.25 HCD = 1.50	LCD = 35 HCD = 38	age 65 to 74 years, Ca > 2.37 mmol/L, iPTH < 100 pg/mL	12 months	Ca carbonate, calcitriol	LCD = 2.35 ± 0.17 mmol/L, HCD = 2.58 ± 0.20 mmol/L, corrected Ca, p < 0.01	LCD = 1.68 ± 0.22 mmol/L, HCD = 2.11 ± 0.27 mmol/L, p < 0.01	LCD = 121.62 ± 33.82, HCD = 68.64 ± 10.23, p < 0.01
Spasovski, 2007	LCD = 1.25 HCD = 1.75	LCD = 26 HCD = 26	ABD, iPTH < 100 pg/mL	6 months	Ca carbonate	LCD = 2.50 ± 1.02 mmol/L, HCD = 2.54 ± 0.26 mmol/L, corrected Ca, n.s.	LCD = 1.48 ± 0.46 mmol/L, HCD = 1.58 ± 0.45 mmol/L, n.s.	LCD = 78.6 ± 44.7, HCD = 53.8 ± 29.6, p < 0.05
Holgado, 2000	LCD = 1.125 or 1.25 HCD = 1.50 or 1.75	LCD = 11 HCD = 10	diabetes, iPTH < 300 pg/mL	12 months	Ca carbonate	LCD = 8.99 ± 0.42 mg/dL*, HCD = 9.49 ± 0.15 mg/dL*, not corrected Ca, n.s.	LCD = 5.40 ± 0.34 mg/dL*, HCD = 5.35 ± 0.34 mg/dL*, n.s.	LCD = 252 ± 59*, HCD = 109 ± 28*, p = 0.04
Sánchez, 2000	LCD = 1.25 HCD = 1.50	LCD = 11 HCD = 9	iPTH < 120 pg/mL	12 months	Ca carbonate	values not available, n.s.	values not available, n.s.	LCD = 99 ± 69, HCD = 79 ± 5, p value not available

Abbreviations: ABD = adynamic bone disease; Ca = calcium; CI = confidence interval; HCD = high calcium dialysate; iPTH = intact parathyroid hormone; IR = interquartile ranges; LCD = low calcium dialysate; n.s. = not significant; P = phosphate; RCT = randomized controlled trial; SD = standard deviation; SE = standard error. *Mean ± SE.

### A meta-analysis of the levels of serum Ca, P and intact PTH

**Figure 3:**
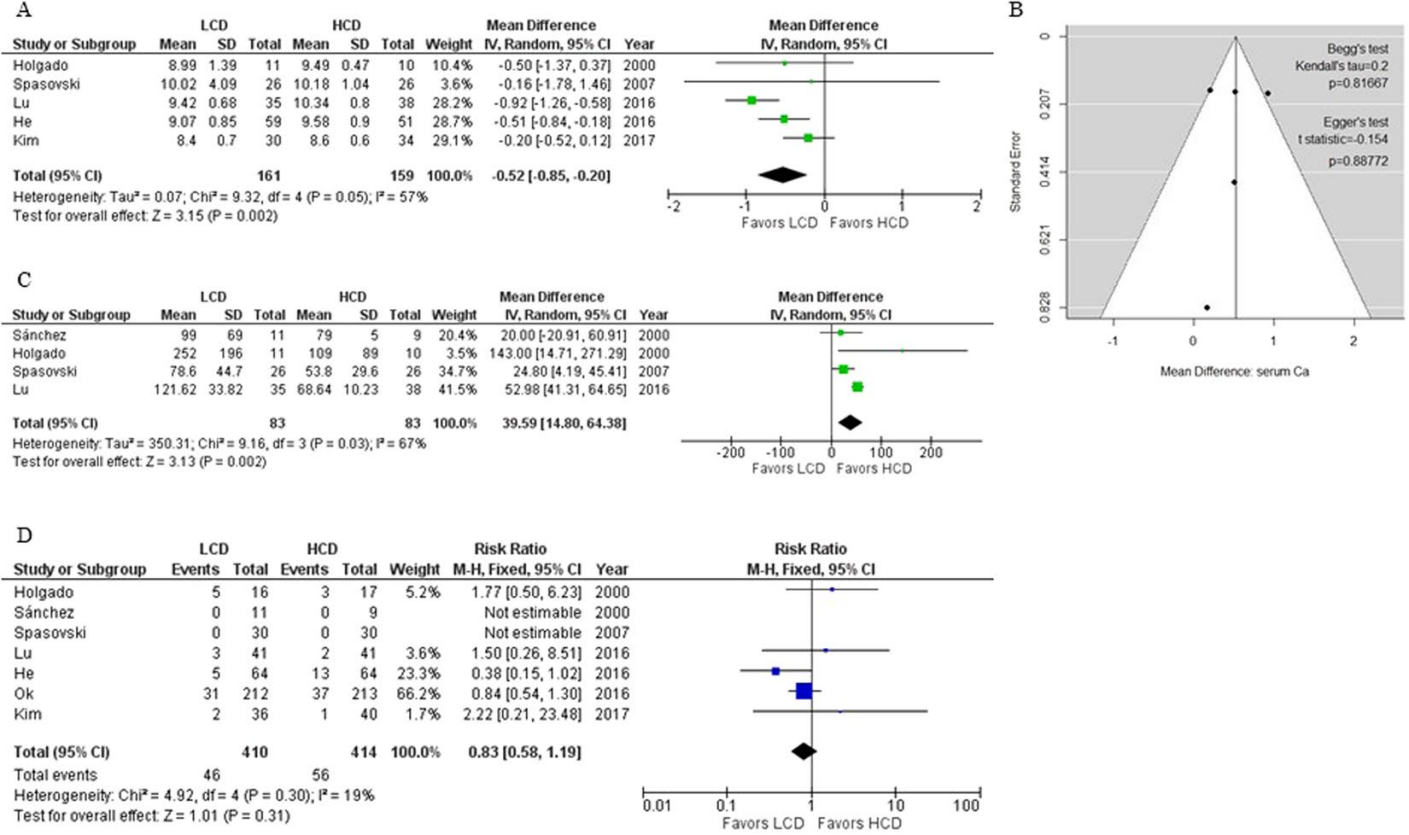
(**A**) Forest plot of mean differences in predialysis serum Ca levels between patients in LCD and HCD groups. (**B**) Funnel plot, Begg’s test and Egger’s test of mean differences in predialysis serum Ca levels between patients in LCD and HCD groups. (**C**) Forest plot of mean differences in serum iPTH levels between patients in LCD and HCD groups. (**D**) Forest plot of risk ratio for mortality between patients in LCD and HCD groups.

First, we aimed to conduct a meta-analysis of the levels of serum Ca, P and intact PTH (iPTH) among patients in all seven RCTs. However, data on the serum Ca and P levels were not available for one RCT^16^, though the data was presented graphically. Another study^12^ only reported the values as time-averaged data during the 24-month study. We excluded these two studies from our meta-analysis of the serum Ca levels. A forest plot of the mean difference (MD) in the predialysis serum Ca levels between patients in the LCD and HCD groups in the other five RCTs is shown in Fig. 3A. This meta-analysis showed that the use of LCD significantly lowered the serum Ca levels, compared with the use of HCD, by 0.52 mg/dL (95% confidence interval [CI], 0.20–0.85; I² = 57%). A funnel plot, Begg’s test^19^ and Egger’s test^20^ showed that a publication bias was not present in this meta-analysis (p > 0.10, Fig. 3B). We could not combine the serum P levels obtained in the five RCTs together for a meta-analysis because of the high heterogeneity (I² = 88%), nor could we observe any tendencies (data not shown). Regarding the serum iPTH levels, three of the seven RCTs^10–12^ showed their values as geometric means (95% CIs), medians (interquartile ranges), and time-averaged data during the 24-month study, respectively. A forest plot of the MD of the serum iPTH levels between patients in the LCD and HCD groups in the other four RCTs is shown in Fig. 3C. This meta-analysis showed that the use of LCD significantly increased the serum iPTH levels, compared with the use of HCD, by 39.59 pg/mL (95% CI, 14.80–64.38; I² = 67%). As a matter of fact, the use of LCD increased the serum iPTH levels in most of the RCTs despite the heterogeneity in the clinical backgrounds, as shown in Table 1.

### Review of the effects on CVD and CKD-MBD

Next, we attempted to review the effects of dCa on CVD and CKD-MBD in patients undergoing maintenance HD. However, the outcomes were shown using different indexes in each study, preventing the data from being combined in a meta-analysis. Instead, we have summarized the study outcomes in Table 2. For CVD, three study groups independently showed that the use of LCD was favorable for preventing the progression of arterial calcifications and/or atherosclerosis in different arteries. He *et al*.^11^ reported that the carotid intima-media thickness (cIMT) and the carotid-femoral pulse wave velocity (cf-PWV), parameters of atherosclerosis and aortic stiffness respectively, were significantly lower in an LCD group than in an HCD group (p = 0.029 and p = 0.024 respectively). Ok *et al*.^12^ showed that the progression rate of the coronary artery calcification scores (CACS) was significantly slower in an LCD group than in an HCD group (p = 0.03). Lu *et al*.^13^ showed that the cIMT, the resistance index and the abdominal aortic calcification scores were notably decreased after 6 and 12 months of study in an LCD group (p < 0.05). However, Kim *et al*.^10^ reported a contradictory outcome, with the CACS increasing in both groups but with the increase in the LCD group being especially significant. As for CKD-MBD, two study groups reported contradictory outcomes. Ok *et al*.^12^ showed that both the bone turnovers and volumes were significantly higher in an LCD group than in an HCD group at 24 months, based on the results of iliac crest bone biopsies. On the other hand, Sánchez *et al*.^16^ reported that the bone mineral density of the lumbar spine was significantly reduced after one year of HD with LCD, but not with HCD, as shown using quantitative computed tomography.

**Table 2:** CVD, CKD-MBD and mortality outcomes of included RCTs.

Author, year	CVD	CKD-BMD	Mortality
Kim, 2017	CACS increased in both groups, especially in the LCD group.	ALP (U/L) at 12 months (mean ± SD): LCD = 90.4 ± 32.1; HCD = 80.2 ± 33.0; p = 0.2217.	2 of 36 died in the LCD group, 1 of 40 died in the HCD group.
He, 2016	cIMT (p = 0.029) and cf-PWV (p = 0.024), parameters of atherosclerosis and aortic stiffness, respectively, were significantly lower in the LCD group than in the HCD group.	No data.	5 of 64 died in the LCD group, 13 of 64 died in the HCD group. Kaplan-Meier curve revealed significancy. p = 0.041.
Ok, 2016	The progression rate of CACS was significantly slower in the LCD group than in the HCD group.	Iliac crest bone biopsies showed that both bone turnovers and volumes were significantly higher in the LCD group than in the HCD group at 24 months. P < 0.05. ALP (U/L) shown as time-averaged data during 24 months (mean ± SD): LCD = 139 ± 72; HCD = 101 ± 52; p = 0.2217.	31 of 212 died in the LCD group, 37 of 213 died in the HCD group.
Lu, 2016	The average IMT of the carotid artery, RI and AACS scores were notably decreased at 6 and 12 months in the LCD group.	No data.	3 of 41 died in the LCD group, 2 of 41 died in the HCD group.
Spasovski, 2007	No data.	ALP (U/L) at 6 months (mean ± SD): LCD = 84.0 ± 35.4; HCD = 65.6 ± 25.9; p < 0.05. BAP: (U/L) at 6 months (mean ± SD): LCD = 35.6 ± 22.3; HCD = 22.5 ± 9.7; p < 0.05.	Nobody died in either group.
Holgado, 2000	No data.	ALP (IU/L) at 6 months (mean ± SE): LCD = 183 ± 31; HCD = 204 ± 46; n.s.	5 of 16 died in the LCD group, 3 of 17 died in the HCD group.
Sánchez, 2000	No data.	After 1 year of hemodialysis, the BMD of the lumbar spine assessed by QCT was significantly reduced in the LCD group, but not in the HCD group. ALP (IU/L) at 1 year (mean ± SD): LCD = 152 ± 38; HCD = 138 ± 38; p value not available.	Nobody died in either group.

Abbreviations: AACS = abdominal aortic calcification score; ALP = alkaline phosphatase; BAP = bone ALP; BMD = bone mineral density; CACS = coronary artery calcium scores; cf-PWV = carotid-femoral pulse wave velocity; cIMT = increased carotid intima-media thickness; CKD = chronic kidney disease; CVD = cardiovascular disease; HCD = high calcium dialysate; LCD = low calcium dialysate; IMT = intimal media thickness; MBD = mineral bone disorders; QCT = Quantitative Computed Tomography; RCT = randomized controlled trial; RI = resistance index; SD = standard deviation; SE = standard error.

### A meta-analysis of the mortality

Finally, we investigated the mortality rate from any cause during each study period in all seven RCTs. As shown in Fig. 3D, the use of LCD did not increase the mortality rate of patients, compared with the use of HCD (risk ratio [RR], 0.83; 95% CI, 0.58–1.19; I² = 19%).

## Discussion

To our knowledge, this is the first systematic review and meta-analysis of the long-term effects of LCD on the serum Ca, P and iPTH levels and on the risk of CVD and CKD-MBD during conventional maintenance HD treatments. We compared the results of seven RCTs with regard to these issues and found that the use of LCD (1.125 or 1.25 mmol/L) significantly lowered the serum Ca levels and raised the serum iPTH levels by 0.52 mg/dL and 39.59 pg/mL, respectively, compared with HCD (1.50 or 1.75 mmol/L) in patients undergoing maintenance HD for 6 months or longer periods. As shown in Table 1, these changes in most cases typically fell within a range which the KDIGO guideline^8,9^ and/or The Japanese Society for Dialysis Therapy guideline recommend^21^, although cinacalcet was not used in any of the seven included RCTs and vitamin D was not used either in the two RCTs where the baseline serum iPTH levels were below 100 or 120 pg/mL. Consistent with our meta-analysis, a meta-analysis^22^ comparing the long**-**term (12–24 months) clinical effects in patients undergoing peritoneal dialysis (PD) using a dialysate with a dCa of 1.25 or 1.75 mmol/L showed that the serum total Ca levels were lower in the LCD group (MD, 0.09 mmol/L; 95% CI, 0.05–0.13; p < 0.0001) and that the serum iPTH levels were significantly higher in the LCD group. In addition, three of the included RCTs^11–13^ showed favorable long-term effects from the use of LCD on the incidence of CVD, as shown in Table 2. One study group^10^, however, unexpectedly showed a contradictory outcome; in this study, the authors inferred that the increased serum iPTH levels in the LCD group might have influenced the progression of coronary artery calcifications. In another RCT that was not included in the present meta-analysis because the patients were randomized between groups receiving LCD with a dCa of 1.12 or 1.37 mmol/L (both lower than 1.50 mmol/L), LeBoeuf *et al*.^23^ showed that the cf-PWV was higher in the higher dCa group, compared with that in the lower dCa group, supporting the idea that a high dCa might be a risk factor for the progression of aortic stiffness in patients receiving maintenance HD. As for CKD-MBD, two study groups reported contradictory outcomes, as shown in Table 2. We suggest, however, that the use of LCD has also favorable effects on CKD-MBD for the following reasons. First, the serum alkaline phosphatase levels, which were regarded as a marker of the bone turnovers in the included RCTs, tended to be higher in the LCD groups than in the HCD groups, as shown in Table 2. Second, Haris *et al*.^24^ conducted a study in which 51 patients treated with maintenance PD and biopsy-proven adynamic bone disease (ABD) were randomized to treatment with control Ca (1.62 mmol/L) or low Ca (1.0 mmol/L) dialysate over a 16-month period. The study showed that the LCD group experienced a decrease in the serum Ca levels, an increase in the serum PTH levels and a rise in bone formation rates into normal range significantly, but that the control group did not.

The KDIGO Guideline considered that LCD would yield neutral Ca balance^8,9^. Positive Ca balance from the dialysate to the body by use of HCD could lead to low turnover bone disease which is associated with a decreased capacity to buffer extra Ca load^25^, so that not only ABD but also ectopic calcification such as atherosclerosis can be caused^26^. In a meta-analysis^27^, one randomized study^28^ of HD patients (although the patients were not randomized according to dCa) showed that the serum Ca levels did not change significantly in patients who underwent long-frequent nocturnal HD (six times per week, ≥ six hours per session) with a dCa ≥ 1.5 mmol/L for 12 months, compared with those who underwent conventional HD with a dCa < 1.5 mmol/L. This result is probably because in such intensive HD treatments, the serum Ca is lost from the body to the dialysate even if a higher dCa is used.

The serum Ca, P and iPTH levels are important therapeutic targets requiring proper control during maintenance HD treatments, but it is often difficult to achieve this because these parameters interact with each other. Hypercalcemia can cause the vascular calcifications that effect the risk of death^29^, while mild hypocalcemia without clinical symptoms is thought to be acceptable^9^. Severe HPT can increase the risk of bone fractures^30^, but the relatively low serum iPTH levels can lead to the low bone turnovers or ABD^31^. Although non-Ca-based P-binders and cinacalcet have been available recently, these medications have also demerits (e.g., side effects such as gastrointestinal distress and binding of essential nutrients)^9^. Moreover, EVOLVE trial^32^ failed to show that cinacalcet reduced the risk of death or major cardiovascular events in patients undergoing HD with moderate-to-severe secondary HPT. Therefore, a consensus remains to be achieved as to which of vitamin D or cinacalcet should be used to prevent secondary HPT, and both of these medications are often used simultaneously^9^. Although additional randomized studies might be needed to determine which dialysate is optimal for preventing the progression of CVD and/or CKD-MBD, our systematic review and meta-analysis suggested that the use of LCD could prevent vascular calcifications by lowering the serum Ca levels, maintain bone turnovers by raising the serum iPTH levels properly and might improve the vital prognosis, as shown in Fig. 3D, in patients undergoing maintenance HD.

Our study had several major limitations. First, the number of RCTs was small, and important data were unavailable in some of the RCTs, reducing the validity of the outcomes of the present meta-analysis. Second, the risks of bias of the included studies were judged to be mostly high or unclear, as shown in Fig. 2. For example, it may be difficult to blind medical doctors to knowledge regarding which dCa their patients received during long-term HD treatment when evaluating performance bias in these kinds of RCTs. Third, the serum Ca levels were corrected according to the serum albumin levels in some RCTs, but not in others, as shown in Table 1. Finally, the present meta-analysis had a moderate or high level of heterogeneity probably because of the variety in clinical backgrounds of the patients included in each RCT, as shown in Table 1. This factor might have led to heterogeneities in the present meta-analysis that cannot be ignored.

In conclusion, the present meta-analysis shows that the use of a low dCa (1.125 or 1.25 mmol/L) significantly reduces the serum Ca levels and raises the serum iPTH levels, compared with the use of a high dCa (1.50 or 1.75 mmol/L), in patients undergoing conventional maintenance HD for 6 months or longer. The use of a low dCa seems to have favorable effects on the risk of CVD in these patients, but additional RCTs are needed to determine which dialysate is optimal for preventing the progression of CVD and/or CKD-MBD among patients receiving long-term conventional maintenance HD treatments.

## Methods

### Patients, interventions and outcomes

We compared the clinical outcomes of RCTs examining patients undergoing conventional maintenance HD for at least 6 months using either a dCa of 1.25 mmol/L or lower or a dCa of 1.50 mmol/L or higher. A total of 622 patients (n = 322 in the LCD group, n = 300 in the HCD group) were included in the present systematic review. Our primary outcomes were the mean differences in the serum Ca, P and iPTH levels at the end of each trial, and our secondary outcomes were the occurrences of CVD, CKD-MBD and deaths from any causes during each trial period.

### Search strategy and eligible criteria

We searched for eligible studies among all papers published prior to July 31, 2017, without any language restrictions using the PubMed and Cochrane Central Register of Controlled Trials (CENTRAL) databases, in accordance with the Preferred Reporting Items for Systematic Reviews and Meta-Analyses (PRISMA) guidelines^33^. Two authors (M.Y. and K.H.) performed the database searches independently. Each discrepancy was discussed until a consensus was reached. Our eligibility criteria were as follows: (1) the study was designed as an RCT; (2) the participants were randomized between an LCD group (dCa of 1.125–1.25 mmol/L) and an HCD group (dCa of 1.50–1.75 mmol/L); (3) the participants were undergoing conventional maintenance HD; and (4) the study period was 6 months or longer. Our terms for the PubMed database search were as follows: (“hemodialysis” [All Fields] OR “renal dialysis” [MeSH Terms] OR (“renal” [All Fields] AND “dialysis” [All Fields]) OR “renal dialysis” [All Fields] OR “hemodialysis” [All Fields]) AND (“dialysis solutions” [Pharmacological Action] OR “dialysis solutions” [MeSH Terms] OR (“dialysis” [All Fields] AND “solutions” [All Fields]) OR “dialysis solutions” [All Fields] OR “dialysate” [All Fields]) AND (“calcium” [MeSH Terms] OR “calcium” [All Fields]) AND (“parathyroid hormone” [MeSH Terms] OR (“parathyroid” [All Fields] AND “hormone” [All Fields]) OR “parathyroid hormone” [All Fields]). For the CENTRAL database search, the terms “hemodialysis, dialysate, calcium” were used. The results of our search process are shown in Fig. 1.

### Data extraction

From the included RCTs, we extracted the following necessary information: first author’s name; publication year; dCa; number of patients; baseline characteristics of patients; study period; medications used during the study period; levels of serum Ca, P and iPTH at the end of the study; surrogate markers (if any) for CVD and CKD-MBD; and mortality during the study period.

### Risk of bias

The risk of bias for each eligible RCT was assessed using the Cochrane Collaboration’s tool^18^ and was categorized as a high, low or unclear bias by two authors (M.Y. and K.H.) acting independently, as shown in Fig. 2. Each discrepancy was discussed until a consensus was reached.

### Data analysis and statistics

The meta-analyses were conducted using random-effects models because the presence of heterogeneity across the RCTs was expected. The heterogeneity was estimated using the I² statistic^33^ and was categorized as low if I² was 0–25%, moderate if I² was 25–75%, or high if I² was 75–100%^34^. The MD and RR with 95% CI data were pooled, and forest plots were drawn using Review Manager, version 5.3. The Begg’s and Egger’s tests^19,20^ were conducted, and a funnel plot was drawn using R software, version 3.4.0 (using the reiv function in the metaphor package), to assess the publication bias. A p value below 0.1 was considered statistically significant in both the Begg’s and Egger’s tests. The units for the serum Ca and P levels were unified using the following formulas: mg/dL × 0.2495 = mmol/L for Ca; mg/dL × 0.3229 = mmol/L for P. The standard error (SE) was converted to the standard deviation (SD) using the following formula: ${\rm{SE}}={\rm{SD}}/\sqrt{{\rm{n}}}$, where n means the sample size of the study.

### Data availability statement

All data analyzed during this study are available on reasonable request to the corresponding author.

## References

[1] Nolan CR (2005). Strategies for improving long-term survival in patients with ESRD. J Am Soc Nephrol..

[2] McCarley PB, Arjomand M (2008). Mineral and bone disorders in patients on dialysis: physiology and clinical consequences. Nephrol Nurs J..

[3] de Jager DJ, Vervloet MG, Dekker FW (2014). Noncardiovascular mortality in CKD: an epidemiological perspective. Nature Reviews Nephrology..

[4] Fournier A (1992). Use of alkaline calcium salts as phosphate binder in uremic patients. Kidney Int..

[5] Patel TV, Singh AK (2009). Role of vitamin D in chronic kidney disease. Semin Nephrol..

[6] Spasovski G (2015). Advances in pharmacotherapy for hyperphosphatemia in renal disease. Expert Opin Pharmacother..

[7] Shahapuni I (2005). How do calcimimetics fit into the management of parathyroid hormone, calcium, and phosphate disturbances in dialysis patients?. Semin Dial..

[8] Gotch FA, Kotanko P, Thijssen S, Levin NW (2010). The KDIGO guideline for dialysate calcium will result in an increased incidence of calcium accumulation in hemodialysis patients. Kidney Int..

[9] Ketteler M (2017). Executive summary of the 2017 KDIGO Chronic Kidney Disease-Mineral and Bone Disorder (CKD-MBD) Guideline Update: what’s changed and why it matters. Kidney Int..

[10] Kim SJ, Lee YK, Oh J, Cho A, Noh JW (2017). Effects of low calcium dialysate on the progression of coronary artery calcification in hemodialysis patients: An open-label 12-month randomized clinical trial. Int J Cardiol..

[11] He Z (2016). Effects of Lowering Dialysate Calcium Concentration on Carotid Intima-Media Thickness and Aortic Stiffness in Patients Undergoing Maintenance Hemodialysis: A Prospective Study. Blood Purif..

[12] Ok E (2016). Reduction of Dialysate Calcium Level Reduces Progression of Coronary Artery Calcification and Improves Low Bone Turnover in Patients on Hemodialysis. J Am Soc Nephrol..

[13] Lu JR (2016). The Study of Low Calcium Dialysate on Elderly Hemodialysis Patients with Secondary Hypoparathyroidism. Blood Purif..

[14] Spasovski G (2007). Improvement of bone and mineral parameters related to adynamic bone disease by diminishing dialysate calcium. Bone..

[15] Holgado R (2000). Effect of a low calcium dialysate on parathyroid hormone secretion in diabetic patients on maintenance hemodialysis. J Bone Miner Res..

[16] Sánchez Perales MC (2000). Hemodialysis with 2.5 mEq/L of calcium in relative hypoparathyroidism: long-term effects on bone mass. Nefrologia..

[17] Moher D (2009). Preferred reporting items for systematic reviews and meta-analyses: the PRISMA statement. PLoS Med..

[18] Higgins JP (2011). The Cochrane Collaboration’s tool for assessing risk of bias in randomised trials. BMJ..

[19] Begg CB, Mazumdar M (1994). Operating characteristics of a rank correlation test for publication bias. Biometrics..

[20] Egger M, Davey Smith G, Schneider M, Minder C (1997). Bias in meta-analysis detected by a simple, graphical test. BMJ..

[21] Tentori F (2015). Recent Changes in Therapeutic Approaches and Association with Outcomes among Patients with Secondary Hyperparathyroidism on Chronic Hemodialysis: The DOPPS Study. Clin J Am Soc Nephrol..

[22] Cao XY (2015). Long term effects on mineral and bone metabolism by low versus standard calcium dialysate in peritoneal dialysis: a meta-analysis. Int J Clin Exp Med..

[23] LeBoeuf A (2011). Impact of dialysate calcium concentration on the progression of aortic stiffness in patients on haemodialysis. Nephrol Dial Transplant..

[24] Haris A, Sherrard DJ, Hercz G (2006). Reversal of adynamic bone disease by lowering of dialysate calcium. Kidney Int..

[25] Kurz P (1994). Evidence for abnormal calcium homeostasis in patients with adynamic bone disease. Kidney Int..

[26] London GM (2004). Arterial calcifications and bone histomorphometry in end-stage renal disease. J Am Soc Nephrol..

[27] Zimmerman DL (2013). Dialysate calcium concentration and mineral metabolism in long and long-frequent hemodialysis: a systematic review and meta-analysis for a Canadian Society of Nephrology clinical practice guideline. Am J Kidney Dis..

[28] Daugirdas JT (2012). Effects of frequent hemodialysis on measures of CKD mineral and bone disorder. J Am Soc Nephrol..

[29] Moldovan D (2016). Mineral and bone disorders, morbidity and mortality in end-stage renal failure patients on chronic dialysis. Clujul Med..

[30] Jadoul M (2006). Incidence and risk factors for hip or other bone fractures among hemodialysis patients in the Dialysis Outcomes and Practice Patterns Study. Kidney Int..

[31] Barreto FC (2008). K/DOQI-recommended intact PTH levels do not prevent low-turnover bone disease in hemodialysis patients. Kidney Int..

[32] EVOLVE (2012). Trial Investigators. Effect of cinacalcet on cardiovascular disease in patients undergoing dialysis. N Engl J Med..

[33] Liberati A (2009). The PRISMA statement for reporting systematic reviews and meta-analyses of studies that evaluate healthcare interventions: explanation and elaboration. BMJ..

[34] Higgins JP, Thompson SG, Deeks JJ, Altman DG (2003). Measuring inconsistency in meta-analyses. BMJ..

